# 新辅助免疫联合化疗在驱动基因阳性非小细胞肺癌患者中的潜在应用价值

**DOI:** 10.3779/j.issn.1009-3419.2024.101.24

**Published:** 2024-09-20

**Authors:** Zihan WEI, Yu ZHOU, Xingxiang PU, Xiang YAN

**Affiliations:** ^1^100871 北京，北京大学人民医院胸外科; ^1^Department of Thoracic Oncology, Peking University People’s Hospital, Beijing 100871, China; ^2^400013 长沙，湖南省肿瘤医院胸部内二科; ^2^The Second Department of Thoracic Oncology, the Affiliated Cancer Hospital of Xiangya School of Medicine, Central South University/Hunan Cancer Hospital, Changsha, 400013, China

**Keywords:** 肺肿瘤, 新辅助免疫治疗, 驱动基因阳性, Lung neoplasms, Neoadjuvant immunotherapy, Diver gene mutation

## Abstract

**背景与目的:**

我国非小细胞肺癌（non-small cell lung cancer, NSCLC）患者中携带驱动基因突变的比例很高，而这部分患者当前的新辅助治疗方案缺乏明显获益。本研究通过分析新辅助免疫联合化疗在驱动基因阳性NSCLC患者中的疗效及不良反应，探索其潜在应用价值。

**方法:**

本研究为双中心回顾性研究，首先纳入来自北京大学人民医院（Peking University People’s Hospital, PKUPH）的在新辅助治疗后手术的驱动基因阳性NSCLC患者，分为单纯化疗组、免疫治疗组以及靶向治疗组三组，比较其疗效及不良反应；其后补充了来自湖南省肿瘤医院（Hunan Cancer Hospital, HNCA）的接受新辅助免疫联合化疗的表皮生长因子受体（epidermal growth factor receptor, EGFR）敏感突变患者来进一步探究EGFR敏感亚型之间对新辅助免疫联合化疗的反应。

**结果:**

共纳入来自两个中心的患者50例。PKUPH的40例患者中采用免疫治疗的有21例，影像学疗效为部分缓解的占比57.1%。病理缓解方面，免疫治疗组的主要病理缓解（major pathological response, MPR）率为38.1%，且仅有免疫治疗组出现了达到完全病理缓解（pathological complete response, pCR）的患者。不同治疗方式之间的不良反应发生率及对手术难度的影响未见显著差异。进一步纳入来自HNCA的10例患者，针对不同新辅助治疗方式下不同EGFR敏感亚型的疗效差异进行进一步分析。新辅助免疫联合化疗与新辅助靶向治疗之间未见显著的影像学缓解差异。而在病理缓解方面，L858R患者仅在接受免疫治疗后出现了MPR、pCR患者，优于靶向治疗患者；19del患者中尚未观察到显著差异。

**结论:**

在不增加不良反应的前提下，新辅助免疫联合化疗取得了更好的MPR、pCR率且远期生存不劣于靶向治疗。

## Abstract (en)

**Background and objective:**

The proportion of patients carrying driver gene mutations is notably high among individuals with non-small cell lung cancer (NSCLC) in China. However, the current neoadjuvant treatment strategies for these patients lack evident benefits. This study aims to investigate the efficacy and adverse reactions of neoadjuvant immunochemotherapy in patients with driver gene-positive NSCLC, thereby exploring its potential therapeutic value.

**Methods:**

A total of 50 patients from two centers were retrospectively collected to compare the efficacy and adverse reactions among driver gene-positive NSCLC patients after different treatments and further explore the response to neoadjuvant immunochemotherapy among different EGFR-sensitive subtypes.

**Results:**

A total of 50 patients from two centers were included in this study. Among the 40 patients from Peking University People’s Hospital (PKUPH), 21 received neoadjuvant immunotherapy, with 57.1% showing partial response on imaging. The major pathological response (MPR) rate after neoadjuvant immunochemotherapy was 38.1%, and pathological complete response (pCR) was only observed in this group. No significant differences were noted in adverse events or their impact on surgical difficulty among different treatments. Additionally, 10 patients from Hunan Cancer Hospital (HNCA) were included to analyze the differences in efficiency among EGFR-sensitive subtypes under various neoadjuvant strategies. No significant radiological response differences were observed between neoadjuvant immunotherapy and targeted therapy. However, patients with the L858R mutation exhibited MPR and pCR only after receiving immunotherapy, surpassing targeted therapy outcomes, while no significant differences were found among 19del patients.

**Conclusion:**

Under the premise of not exacerbating adverse effects, neoadjuvant immunochemotherapy achieved superior rates of MPR and pCR, with long-term survival comparable to targeted therapy.

据GLOBOCAN2022公布的数据^[1]^，肺癌在发病率上超越了乳腺癌，成为全球最常见癌症，且成为癌症相关死亡的首要原因，在中国人群中亦是如此^[2]^，其中80%-85%为非小细胞肺癌（non-small cell lung cancer, NSCLC）。与欧美人群不同，我国NSCLC呈现出驱动基因阳性占比较高的特点，尤以表皮生长因子受体（epidermal growth factor receptor, EGFR）突变为著，40.0%-57.7%的NSCLC包含EGFR基因突变^[3,4]^。

肺癌的首选治疗措施是手术切除，但单纯手术治疗疗效并不满意，其原因主要集中在局部复发及远处转移方面。因此，指南推荐肺癌最佳治疗方式为以手术为基础的围术期综合治疗^[5]^。相较于辅助治疗缺乏可测量病灶及有效的疗效检测方式，新辅助治疗可直接通过观察病灶变化来判断药物敏感性，同时具备缩小病灶大小、降低手术难度、改善患者预后等优势。针对驱动基因阳性的可切除NSCLC患者，目前临床在新辅助治疗阶段仍主要采用新辅助化疗或靶向治疗的方式，然而其并未取得令人满意的疗效，其中新辅助化疗的完全病理缓解（pathological complete response, pCR）率大致在10%^[6⇓⇓-9]^，而综合CTONG1103、Neo-ADAURA等已发表的数据显示，新辅助靶向治疗的pCR率波动在0%-12.1%^[10⇓⇓⇓-14]^。

随着免疫治疗的进一步发展，NADIM、KEYNOTE-671等部分临床试验^[15⇓-17]^中纳入的少数驱动基因阳性患者获得了较好的疗效，体现出了新辅助免疫治疗在驱动基因阳性患者中的应用潜力，但前述研究中驱动基因阳性患者病例数较少。本研究通过对比真实世界中驱动基因阳性患者不同新辅助治疗方式的疗效，进一步探究新辅助免疫治疗应用于驱动基因阳性可切除NSCLC患者的可行性。

## 1 资料与方法

### 1.1 临床资料

本研究为双中心回顾性队列研究（图1）。回顾2018年1月1日至2023年10月1日就诊于北京大学人民医院（Peking University People’s Hospital, PKUPH）行新辅助治疗的I-IIIB期[依据美国肿瘤联合会（American Joint Committee on Cancer, AJCC）第8版肺癌肿瘤原发灶-淋巴结-转移（tumor-node-metastasis, TNM）分期标准]NSCLC患者。纳入标准：（1）经活检病理证实存在驱动基因突变（至少检测EGFR）；（2）经多学科联合会诊（multidisciplinary team, MDT）团队评估后接受新辅助治疗；（3）新辅助治疗后接受根治性手术切除。排除标准：（1）临床资料不完整；（2）无驱动基因阳性突变结果。

**图1:**
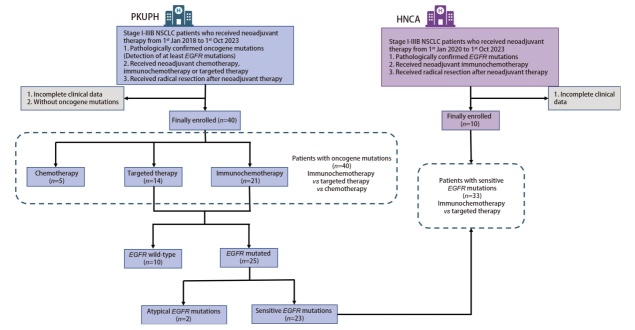
患者入组流程

分中心补充队列：回顾2020年1月1日至2023年10月1日就诊于湖南省肿瘤医院（Hunan Cancer Hospital, HNCA）行新辅助免疫联合化疗的I-IIIB期NSCLC患者。纳入标准：（1）经活检病理证实存在EGFR突变；（2）经MDT团队评估后接受新辅助免疫联合化疗；（3）新辅助治疗后接受根治性手术切除。排除标准：临床资料不完整。

本研究涉及的驱动基因包括EGFR、Kirsten大鼠肉瘤病毒癌基因同源物（Kirsten rat sarcoma viral oncogene homolog, KRAS）、酪氨酸激酶受体（Erb-b2 receptor tyrosine kinase 2, ERBB2）、间质表皮转化因子（mesenchymal to epithelial transition factor, MET）、RAF家族丝氨酸/苏氨酸蛋白激酶（B-Raf proto-oncogene, serine/threonine kinase, BRAF）、间变性淋巴瘤激酶（anaplastic lymphoma kinase, ALK）融合变异、c-ROS肉瘤致癌因子-受体（ROS proto-oncogene 1-receptor, ROS1）融合变异、原癌基因酪氨酸蛋白激酶受体（proto-oncogene tyrosine-protein kinase receptor, RET）融合变异，其中EGFR分为敏感突变（包括19del及L858R）及罕见突变（其他亚型）。入组患者要求至少包含上述一种基因变异。研究涉及的其余合并非驱动基因变异包括TP53、MUC16、RBM10、PIK3CA、PTEN。

本文为回顾性研究，经北京大学人民医院伦理审查委员会审查通过后开展并免除患者知情同意（伦理批件号：2023PHD008-001）。

### 1.2 治疗方法

依据新辅助治疗方案将我中心患者分为3组。（1）单纯化疗组：患者均采用以铂类为基础的双药化疗，包括培美曲塞、吉西他滨、白蛋白紫杉醇等；（2）免疫治疗组：经MDT团队评估后予以程序性死亡受体/配体1（programmed cell death 1/programmed cell death ligand 1, PD-1/PD-L1）靶点免疫抑制剂新辅助治疗，并同期给予含铂双药化疗治疗；（3）靶向治疗组：依据患者驱动基因类型经MDT团队评估后给予相应靶向治疗药物，可同期给予或不给予含铂双药化疗。

分中心补充队列患者均接受新辅助免疫联合化疗，方案同免疫治疗组。

免疫治疗组患者每2个周期评效，经MDT讨论后决定是否继续1-2个周期治疗后手术；靶向治疗组患者依据其影像学缓解程度每1-3个月评效；手术为末次治疗4-8周后。术后辅助治疗依据手术大病理结果经MDT讨论决定。

### 1.3 主要观察指标及随访

主要从疗效及并发症发生率两个角度来衡量。疗效方面关注患者新辅助治疗后影像学缓解率[依据《实体瘤疗效评价标准》分为完全缓解（complete response, CR）、部分缓解（partial response, PR）、疾病稳定（stable disease, SD）、疾病进展（progressive disease, PD）]、病理学缓解[包括主要病理缓解（major pathological response, MPR）和pCR]、无病生存期（disease-free survival, DFS）及总生存期（overall survival, OS）等。并发症关注新辅助治疗阶段[依据不良事件常用术语标准（Common Terminology Criteria for Adverse Events, CTCAE）]、手术相关并发症两个方面。随访通过门诊复诊或电话联系进行，患者术后第1年内每3个月复查胸部计算机断层扫描（computed tomography, CT），随后2-5年内每半年复查胸部CT，头颅核磁、腹部超声、骨扫描等检查每年1次，或者根据医生的判断必要时进行。随访截止时间为2024年2月5日。

### 1.4 统计学方法

采用SPSS 26.0处理数据，计数资料以频数（%）表示，本研究两部分组间比较总体样本量≤40例，故组间比较采用费舍尔精确检验（Fisher’s exact test）。计量资料符合正态分布时，以均数±标准差（Mean±SD）表示，两组间比较采用独立样本t检验，三组间比较采用方差分析（ANOVA）；不符合正态分布时用中位数结合极值[Median (Min, Max)]的形式描述，并使用Wilcoxon秩和检验进行两组间比较，使用Kruskal-Wallis秩和检验进行三组间比较。采用Kaplan-Meier法绘制DFS、OS曲线，组间比较采用Log-rank检验。以P<0.05为差异有统计学意义。

## 2 结果

### 2.1 患者基本特征

PKUPH共入组40例患者（图1），其中单纯化疗组5例，靶向治疗组14例，免疫治疗组21例。男性占比50%，患者平均年龄59.4岁，72.5%的患者从未吸烟，29例（72.5%）患者在新辅助治疗前为III期，26例初诊时淋巴结分期为N2，29例患者病理类型为腺癌。驱动基因突变方面，以EGFR为著，共27例，占比67.5%，其中敏感突变（19del、L858R）占比62.5%；其余驱动基因突变特征还包括ALK融合（5例，12.5%）、BRAF（1例，2.5%）、HER2（1例，2.5%）、KRAS（2例，5.0%）、RET融合（2例，5.0%）、ROS1融合（2例，5.0%）（图2）。共20例患者出现合并突变，其中EGFR合并TP53突变5例，TP53、RBM10突变1例，PIK3CA突变2例，RET融合1例，MUC16、MET突变1例，RBM10、PIK3CA突变1例，ROS1融合1例；ALK融合合并TP53、MUC16突变2例；RET融合合并TP53突变1例；KRAS突变合并TP53突变1例；ROS1融合合并TP53突变2例，BRAF合并TP53突变1例，HER2合并TP53突变1例。三种不同治疗方式之间患者性别、年龄、吸烟史、临床分期、治疗周期、PD-L1表达率无显著差异（表1）。

**图2:**
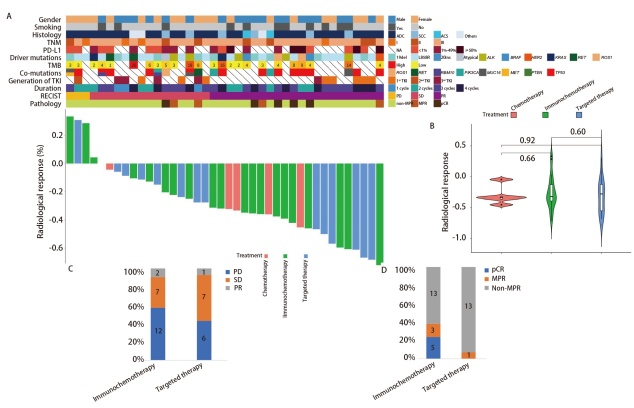
PKUPH驱动基因阳性患者新辅助治疗疗效评价。A：PKUPH驱动基因阳性患者临床病理特征及影像学缩瘤率概览；B：不同新辅助治疗方式患者缩瘤率比较；C：新辅助免疫治疗与靶向治疗患者影像学缓解率比较；D：新辅助免疫治疗与靶向治疗患者病理缓解率比较。

**表1:** PKUPH患者基线临床特征

Clinical demographics	All patients	Chemotherapy	Immunochemotherapy	Targeted therapy	P
	(n=40)	(n=5)	(n=21)	(n=14)	
Gender					0.069
Male	20 (50.0%)	2 (40.0%)	14 (66.7%)	4 (28.6%)	
Female	20 (50.0%)	3 (60.0%)	7 (33.3%)	10 (71.4%)	
Age (yr, Mean±SD)	59.4±9.2	52.0±10.7	61.1±8.9	59.5±8.5	0.139
Smoking					0.108
Former	11 (27.5%)	1 (20.0%)	8 (38.1%)	2 (14.3%)	
Never	29 (72.5%)	4 (80.0%)	13 (61.9%)	12 (85.7%)	
EGFR subtype					0.012
L858R	16 (40.0%)	1 (20.0%)	6 (28.6%)	9 (64.3%)	
19del	9 (22.5%)	1 (20.0%)	3 (14.3%)	5 (35.7%)	
20ins	1 (2.5%)	0 (0.0%)	1 (4.8%)	0 (0.0%)	
C.241-8C>T	1 (2.5%)	0 (0.0%)	1 (4.8%)	0 (0.0%)	
Other driver genes	13 (32.5%)	3 (60.0%)	10 (47.6%)	0 (0.0%)	
Histology					<0.001
Adenocarcinoma	29 (72.5%)	4 (80.0%)	12 (57.1%)	13 (92.9%)	
Squamous	6 (15.0%)	0 (0.0%)	6 (28.6%)	0 (0.0%)	
Adenosquamous carcinoma	2 (5.0%)	1 (20.0%)	1 (4.8%)	0 (0.0%)	
Others	3 (7.5%)	0 (0.0%)	2 (9.5%)	1 (7.1%)	
Clinical stage					P_I vs II vs III _=0.444
I	4 (10.0%)	0 (0.0%)	1 (4.8%)	3 (21.4%)	
II	7 (17.5%)	1 (20.0%)	3 (14.3%)	3 (21.4%)	
IIA	3 (7.5%)	1 (20.0%)	2 (9.5%)	0 (0.0%)	
IIB	4 (10.0%)	0 (0.0%)	1 (4.8%)	3 (21.4%)	
III	29 (72.5%)	4 (80.0%)	17 (81.0%)	8 (57.1%)	
IIIA	16 (40.0%)	4 (80.0%)	9 (42.9%)	3 (21.4%)	
IIIB	13 (32.5%)	0 (0.0%)	8 (38.1%)	5 (35.7%)	
Duration*					0.432
1-2 cycle(s)	13 (32.5%)	4 (80.0%)	4 (19.0%)	5 (35.7%)	
3-4 cycles	27 (67.5%)	1 (20.0%)	17 (81.0%)	9 (64.3%)	
Adjuvant treatment					<0.001
Chemotherapy	2 (5.0%)	2 (40.0%)	0 (0.0%)	0 (0.0%)	
Immunochemotherapy	12 (30.0%)	1 (20.0%)	11 (52.4%)	0 (0.0%)	
Targeted therapy	19 (47.5%)	2 (40.0%)	5 (23.8%)	12 (85.7%)	
None	7 (17.5%)	0 (0.0%)	5 (23.8%)	2 (14.3%)	
PD-L1					0.071
<1%	10 (25.0%)	1 (20.0%)	6 (28.6%)	3 (21.4%)	
1%-49%	8 (20.0%)	0 (0.0%)	8 (38.1%)	0 (0.0%)	
≥50%	5 (12.5%)	2 (40.0%)	2 (9.5%)	1 (7.1%)	
NA	17 (42.5%)	2 (40.0%)	5 (23.8%)	10 (71.4%)	

*Targeted therapy is administered on a monthly cycle. PD-L1：programmed cell death ligand 1; NA: not available.

PD-L1表达水平被认为与免疫治疗预后相关，在PKUPH的数据中，PD-L1表达水平在不同治疗方式亚组之间无显著差异（P=0.071，图3A）。进一步分析PD-L1表达水平与驱动基因之间的关系发现，PD-L1表达在EGFR突变型及其他驱动基因阳性患者之间并无显著差异（P=0.656，图3B）。值得注意的是，PKUPH的驱动基因阳性患者中，肺腺癌PD-L1阳性率（PD-L1≥1%）与肺鳞癌之间无显著差异（56.25% vs 40.00%, P=0.635），有5例（31.25%）肺腺癌患者为PD-L1高表达（PD-L1≥50%），但与肺鳞癌之间并无统计学差异（P=0.278，图3C）。PD-L1阳性率及高表达率与分期无明显关系（图3D）。

**图3:**
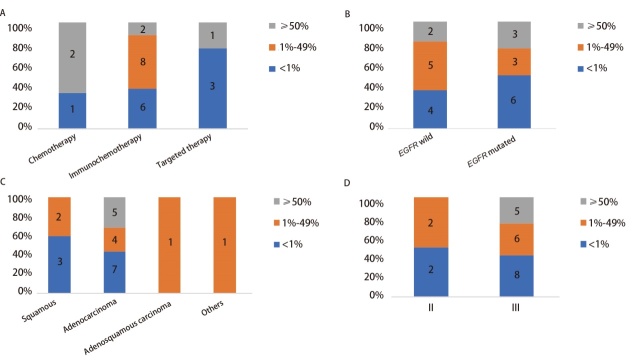
PD-L1表达水平的差异。A：不同新辅助治疗方案；B：不同EGFR突变状态（EGFR野生型患者含有其他驱动基因突变）；C：不同组织学亚型；D：不同临床分期。

### 2.2 疗效评价

PKUPH的驱动基因阳性患者新辅助治疗整体客观缓解率（objective response rate, ORR）为55.0%（图2A）。免疫治疗组影像学疗效为PR的患者为57.1%（12/21），单纯化疗组和靶向治疗组分别为80.0%（4/5）、42.9%（6/14），三种治疗方式的ORR及缩瘤率之间未见显著差异（表2，图2B、2C）。

**表2:** 不同新辅助治疗患者疗效

Items	All patients	Chemotherapy	Immunochemotherapy	Targeted therapy	P
	(n=40)	(n=5)	(n=21)	(n=14)	
Tumor shrinkage (Mean±SD)	-0.285±0.258	-0.302±0.153	-0.262±0.267	-0.313±0.284	0.844
Radiological response					0.746
PR	22 (55.0%)	4 (80.0%)	12 (57.1%)	6 (42.9%)	
SD	15 (37.5%)	1 (20.0%)	7 (33.3%)	7 (50.0%)	
PD	3 (7.5%)	0 (0.0%)	2 (9.5%)	1 (7.1%)	
Pathological response					0.170
pCR	5 (12.5%)	0 (0.0%)	5 (23.8%)	0 (0.0%)	
MPR	4 (10.0%)	0 (0.0%)	3 (14.3%)	1 (7.1%)	
non-MPR	31 (77.5%)	5 (100.0%)	13 (61.9%)	13 (92.9%)	
pCR vs non-pCR					<0.001
pCR	5 (12.5%)	0 (0.0%)	5 (23.8%)	0 (0.0%)	P_immuno vs targeted _=0.069
non-pCR	35 (87.5%)	5 (100.0%)	16 (76.2%)	14 (100.0%)	
MPR vs non-MPR					0.013
MPR	9 (22.5%)	0 (0.0%)	8 (38.1%)	1 (7.1%)	P_immuno vs targeted _=0.056
non-MPR	31 (77.5%)	5 (100.0%)	13 (61.9%)	13 (92.9%)	

PR: partial response; SD: stable disease; PD: progressive disease; MPR: major pathological response; pCR: pathological complete response.

病理缓解方面，免疫治疗组的MPR率为38.1%（8/21），高于单纯化疗组（0.0%, 0/5）及靶向治疗组（7.1%, 1/14），且仅有免疫治疗组出现了治疗后达到pCR的患者（图2D），占比23.8%（5/21）（表2）。进一步比较免疫治疗组及靶向治疗组MPR、pCR组间差异发现，二者并未达到统计学差异。

在中位时长为16个月的随访中，免疫治疗组的远期生存指标不劣于靶向治疗组（DFS: P=0.750; OS: P=0.640）（图4）。其中靶向治疗组的中位DFS为20个月，由于目前随访时间较短，免疫治疗组尚未达到中位DFS；二者均未达到中位OS。

**图4:**
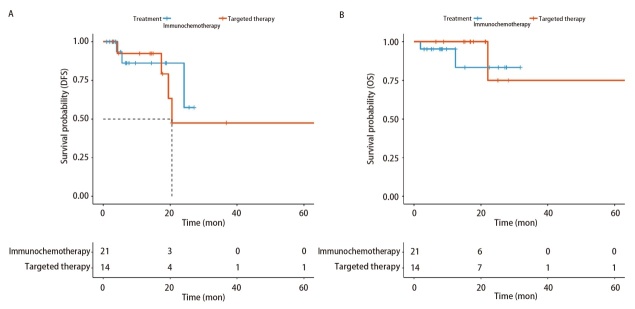
新辅助免疫治疗与靶向治疗远期生存指标对比。A：DFS；B：OS。

### 2.3 不同治疗方式对手术难度的影响

免疫治疗组患者平均手术时长为200 min，略高于靶向治疗组（170 min），二者并无统计学差异（P=0.132）。术中出血量方面，免疫治疗组患者中位术中出血量30 mL，靶向治疗组为50 mL，二者无统计学差异（P=0.451）。有3例接受免疫治疗的患者术中出血量>200 mL并予以术中输血。1例接受新辅助靶向治疗的患者术中出现房颤，后自行转复，术后未见明显不适。免疫治疗组与靶向治疗组患者的总住院时长（12 vs 10.5 d, P=0.905）以及术后住院时长（均为5 d，P>0.999）无显著差异。以手术时长、术中出血量、术中不良反应、住院总时长及术后住院时长指标评估靶向治疗组与免疫治疗组的手术难度，二者并无显著差异（表3）。

**表3:** 不同治疗方式对手术难度的影响

Items	All patients	Chemotherapy	Immunochemotherapy	Targeted therapy	P_immuno vs targeted_
	(n=40)	(n=5)	(n=21)	(n=14)	
Surgical time (min, Mean±SD)	197.0±63.4	258.0±78.0	200.0±61.6	170.0±46.0	0.132
Intraoperative hemorrhage (mL)^#^	50 (10, 1400)	50 (50, 100)	30 (10, 1400)	50 (20, 200)	0.451
Surgery related AEs					0.170
Hemorrhage*	3 (7.5%)	0 (0.0%)	3 (14.3%)	0 (0.0%)	
Intraoperative atrial fibrillation	1 (2.5%)	0 (0.0%)	0 (0.0%)	1 (7.1%)	
Hospitalization (d)^#^	11.0 (7.0, 24.0)	9.0 (7.0, 12.0)	12.0 (8.0, 18.0)	10.5 (8.0, 24.0)	0.905
Hospitalization after surgery (d)^#^	5.0 (3.0, 12.0)	4.0 (3.0, 5.00)	5.0 (3.0, 12.0)	5.0 (3.0, 11.0)	>0.999

*Intraoperative hemorrhage more than 200 mL. ^#^Median (Min, Max). AE: adverse event.

### 2.4 治疗相关不良反应

依据CTCAE 5.0标准，共24例患者出现1-4级不良反应，仅免疫治疗组出现1例4级不良反应，表现为中性粒细胞减少，经入院治疗后恢复正常值。对比靶向治疗组与免疫治疗组，二者新辅助治疗阶段不良反应发生率并无显著差异（78.6% vs 47.6%，P=0.108，表4）。共5例患者新辅助治疗过程中出现了肺部纤维化，均为1级不良反应，5例患者均完成了3-4个周期的新辅助治疗；其中靶向治疗组出现肺部纤维化患者1例，患者为EGFR 19del合并RET融合突变，术后病理达到MPR；免疫治疗组出现肺部纤维化患者4例，其中EGFR罕见突变及HER2突变患者达到了MPR，ALK融合及EGFR L858R患者未达到MPR。其他常见不良反应包括骨髓抑制、肝功能受损等。

**表4:** 不同新辅助治疗相关并发症

Adverse events	All patients	Chemotherapy	Immunochemotherapy	Targeted therapy	P_immuno vs targeted_
	(n=40)	(n=5)	(n=21)	(n=14)	
G1	9 (22.5%)	0 (0.0%)	5 (23.8%)	4 (28.6%)	0.108
G2	6 (15.0%)	3 (60.0%)	0 (0.0%)	3 (21.4%)	
G3	8 (20.0%)	0 (0.0%)	4 (19.0%)	4 (28.6%)	
G4	1 (2.5%)	0 (0.0%)	1 (4.8%)	0 (0.0%)	

### 2.5 EGFR敏感突变不同治疗方式

PKUPH共纳入23例EGFR敏感突变患者，其中L858R 15例、19del 8例；为了增加样本量以获得更有价值的数据结果，本研究纳入了来自HNCA的EGFR突变型新辅助免疫治疗的患者10例。两中心新辅助免疫治疗患者基线特征无显著差异。遂合并为免疫治疗组，与PKUPH的靶向治疗组患者进行比较。不同治疗方式之间患者的性别分布、年龄、吸烟史、美国东部肿瘤协作组（Eastern Cooperative Oncology Group, ECOG）体能状态评分无显著差异。纳入的患者中以III期患者为主，免疫治疗组有14例（73.7%），靶向治疗组有8例（57.1%）。分子特征方面，免疫治疗组纳入9例19del突变型患者（47.4%），10例L858R突变型患者（52.6%）；靶向治疗组纳入5例19del突变型患者（35.7%），9例L858R突变型患者（64.3%）；二者分布无统计学差异（P=0.722）。

在靶向治疗组中，患者应用的靶向药物主要集中在一代及三代靶向药，其中以埃克替尼（7/14, 50.0%）与奥希替尼（4/14, 28.6%）为著，除此之外，分别有1例患者应用了吉非替尼、达克替尼及阿美替尼。靶向治疗组仅有1例应用奥希替尼的患者在新辅助治疗后达到了MPR，其余患者均未达到MPR。

影像学疗效评估显示，免疫治疗组肿瘤直径平均缩小了27.0%，靶向治疗组为31.3%，二者无显著差异（P=0.671）（表5）。患者均未达到影像学CR，免疫治疗组患者达到PR的比例高于靶向治疗组患者（63.2% vs 42.9%），但未达到统计学显著差异。病理学疗效评估显示，仅有免疫治疗组出现了pCR的患者，共4例，占比21.1%；免疫治疗组未达到MPR患者的比例低于靶向治疗组（68.4% vs 92.9%）。综合来看，免疫治疗组的pCR显著优于靶向治疗组（P<0.001）。

**表5:** EGFR敏感突变患者新辅助治疗疗效

Items	Overall	Immunochemotherapy	Targeted therapy	P
	(n=33)	(n=19)	(n=14)	
Tumor shrinkage (Mean±SD)	-0.288±0.284	-0.270±0.291	-0.313±0.284	0.671
Radiological response				0.061
PR	18 (54.5%)	12 (63.2%)	6 (42.9%)	
SD	12 (36.4%)	5 (26.3%)	7 (50.0%)	
PD	3 (9.1%)	2 (10.5%)	1 (7.1%)	
Pathological response				<0.001
pCR	4 (12.1%)	4 (21.1%)	0 (0.0%)	
MPR	3 (9.1%)	2 (10.5%)	1 (7.1%)	
non-MPR	26 (78.8%)	13 (68.4%)	13 (92.9%)	

针对不同新辅助治疗方式下不同EGFR敏感亚型的疗效差异进行进一步分析，携带L858R敏感亚型的患者在接受新辅助免疫联合化疗后的缩瘤率达到了（-0.332±0.303），优于靶向治疗（-0.308±0.318）；携带19del敏感亚型的患者在接受新辅助免疫联合化疗后的缩瘤率达到了（-0.200±0.277），劣于靶向治疗（-0.323±0.245）。不同亚组间影像学疗效评价（图5A）未见显著差异。病理缓解率方面（图5B），L858R突变型患者仅在接受新辅助免疫治疗后达到了MPR、pCR，新辅助靶向治疗患者均未达到MPR，其中MPR率（50.0% vs 0.0%）达到了统计学差异（P=0.033）。19del突变型患者中尚未观察到这类差异。

**图5:**
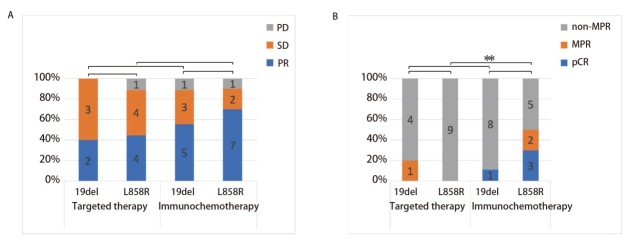
不同治疗方式对EGFR不同敏感亚型NSCLC疗效评价。A：影像学疗效评价；B：病理学疗效评价。

## 3 讨论

这项回顾性研究中纳入了50例真实世界的驱动基因阳性NSCLC患者，评估不同新辅助治疗方式的疗效比较，对影像学、病理学评估做了严格的质量控制。本研究发现，在不增加不良反应的前提下，免疫治疗组相对于靶向治疗组在数值上取得了更好的MPR、pCR率。受限于样本量及随访时间，二者之间的PFS、OS未取得显著差异，但免疫治疗组在趋势上有长期生存的优势。对EGFR患者群体做进一步分析发现，携带EGFR L858R突变的患者在接受新辅助免疫治疗后的MPR率显著高于新辅助靶向治疗，且在数值上高于接受新辅助免疫及靶向治疗的携带EGFR 19del突变的患者。这提示我们EGFR L858R突变型NSCLC患者或许是新辅助免疫治疗的潜在获益人群。

驱动基因阳性患者的新辅助治疗标准为化疗。而新辅助化疗应用临床多年，其pCR率大致稳定在10%^[6⇓⇓-9]^，难以突破治疗瓶颈。研究者们对驱动基因阳性患者的新辅助靶向治疗疗效进行了进一步探索后发现，无论应用EGFR靶向药的代际如何，其pCR率波动于0%-12.1%^[10,12⇓-14]^。Neo-ADAURA研究在进一步完善了研究设计后，已披露的28例患者中，仅1例获得了pCR。而针对其余驱动基因阳性的新辅助靶向治疗仅有少数研究（LCMC4等）正在进行中，尚未得到明确有效的结论。因此，驱动基因阳性患者新辅助治疗阶段的疗效亟待改善。值得注意的是，在少数的病例中，EGFR阳性患者似乎对新辅助免疫治疗展现了良好的治疗效果。KEYNOTE-671^[18]^纳入的患者中，治疗组与对照组分别仅有十余例携带EGFR/ALK变异，与亚洲人群的基因突变频率差别较大，且研究者未展示驱动基因阳性患者的病理缓解率相关数据。研究披露的数据显示，相比较于EGFR野生型患者，EGFR突变型患者在免疫联合化疗新辅助治疗后取得了更长的无事件生存率（event free survival, EFS）。而广东省肺癌研究所发布的针对驱动基因阳性患者采用新辅助免疫治疗的研究^[16,19]^中，pCR率达到了12.5%，这提示我们新辅助免疫治疗在驱动基因阳性患者中的疗效并不弱于新辅助靶向治疗。PKUPH共有23.8%的驱动基因阳性患者在接受新辅助免疫治疗后达到了pCR。除EGFR突变型之外，有10例其他驱动基因阳性患者接受了新辅助免疫治疗，其中2例（20%，分别携带ROS1融合变异合并TP53突变、BRAF突变合并TP53突变）在新辅助免疫治疗后达到了pCR。合并突变一样值得关注，在合并TP53突变的14例患者中，有10例患者接受了免疫治疗，其中5例达到了MPR、3例达到了pCR。由于其他驱动基因阳性患者样本量小，无法得到统计学显著的疗效差异；但从不良反应方面评估，对驱动基因阳性患者采用新辅助免疫治疗并未增加患者不良反应发生率。因此，驱动基因阳性患者的新辅助免疫治疗潜力值得进一步探索。

在携带EGFR突变的患者中，研究人员通过比较肿瘤免疫微环境发现，L858R突变型的肿瘤免疫微环境中CD8^+^ PD-1^+^ T细胞浸润多于19del^[20]^，同时Offin等^[21]^发现L858R突变型患者肿瘤突变负荷（tumor mutational burden, TMB）高于19del。接受免疫单药治疗的晚期NSCLC患者，其研究终点包括ORR在不同的EGFR突变亚型中差异显著，L858R突变型患者的ORR高于19del（20.8% vs 9.5%）。本研究结果显示L858R突变型患者接受新辅助免疫治疗之后病理缓解率优于靶向治疗，其MPR率达到了统计学差异（50.0% vs 0.0%, P=0.033）。同时可以观察到，接受免疫治疗的患者中，L858R突变型患者MPR率在数值上高于19del（50.0% vs 11.0%, P=0.141）。其疗效差异可能源自L858R突变型NSCLC患者具备一个更活跃的免疫微环境。另一方面，L858R突变型NSCLC对靶向治疗反应不佳，FLURA研究评估了携带EGFR突变的晚期NSCLC患者靶向治疗的疗效，发现L858R突变型患者中位PFS相较于19del缩短了7个月（21.4 vs 14.4个月）^[22]^；同时在辅助治疗阶段，ADAURA的亚组分析提示，与安慰剂治疗组相比，L858R突变型患者接受辅助奥希替尼治疗后HR为0.31（95%CI: 0.18-0.49），而19del突变型患者的HR为0.12（95%CI: 0.07-0.20）^[23]^。综合上述研究结果，EGFR L858R突变型患者或许是新辅助免疫治疗的潜在获益人群。由于当前的研究样本量偏小，仍需大型的前瞻性临床试验进一步证实上述结论，进一步优化EGFR突变型可切除NSCLC的新辅助免疫治疗策略。

本研究共纳入4例I期NSCLC患者，为降低手术难度、缩小切除范围，经患者及家属同意后行新辅助治疗。4例患者均携带EGFR L858R突变，其中3例应用新辅助靶向治疗后均达到影像学PR，而1例在应用新辅助免疫治疗后PD；4例患者术后病理均未达到MPR。这些结果提示，在对I期患者应用新辅助治疗时应严格监测患者病灶变化，必要时及时予以手术介入。

作为一个双中心回顾性的研究，这项研究存在一些局限性。首先，研究纳入的样本量偏少，治疗方式不一致、周期不一致。但本研究是目前同一时空条件下样本量较大的有关驱动基因阳性NSCLC新辅助免疫治疗疗效的研究，且数据为真实世界临床数据，能客观反映临床诊疗现状，具有一定的临床价值。其次，研究涉及的具体的新辅助治疗药物选择及治疗周期并不一致，这也影响了研究结果的证据等级。

本研究对新辅助免疫治疗对驱动基因阳性NSCLC的潜在应用价值进行了初步探索，在不增加不良反应的前提下，接受新辅助免疫治疗患者疗效不弱于接受新辅助靶向治疗患者。期待新的大型多中心前瞻性临床研究进一步探索新辅助免疫治疗对该类型患者的远期生存结局，进一步改善这部分患者的远期预后。
